# Limited Effects of Type I Interferons on Kyasanur Forest Disease Virus in Cell Culture

**DOI:** 10.1371/journal.pntd.0004871

**Published:** 2016-08-01

**Authors:** Bradley W. M. Cook, Charlene Ranadheera, Aidan M. Nikiforuk, Todd A. Cutts, Darwyn Kobasa, Deborah A. Court, Steven S. Theriault

**Affiliations:** 1 Applied Biosafety Research Program, National Microbiology Laboratory at the Canadian Science Centre for Human and Animal Health and National Microbiology Laboratory at the J. C. Wilt Infectious Diseases Research Centre, Public Health Agency of Canada, Winnipeg, Manitoba, Canada; 2 Department of Microbiology, The University of Manitoba, Winnipeg, Manitoba, Canada; 3 High Containment Respiratory Viruses Group, Special Pathogens Program, National Microbiology Laboratory at the Canadian Science Centre for Human and Animal Health, Public Health Agency of Canada, Winnipeg, Manitoba, Canada; 4 Department of Medical Microbiology, The University of Manitoba, Winnipeg, Manitoba, Canada; Molecular Biology Unit (MBU), INDIA

## Abstract

**Background:**

The tick-borne flavivirus, Kyasanur Forest disease virus (KFDV) causes seasonal infections and periodic outbreaks in south-west India. The current vaccine offers poor protection with reported issues of coverage and immunogenicity. Since there are no approved prophylactic therapeutics for KFDV, type I IFN-α/β subtypes were assessed for antiviral potency against KFDV in cell culture.

**Methodology/Principal Findings:**

The continued passage of KFDV-infected cells with re-administered IFN-α2a treatment did not eliminate KFDV and had little effect on infectious particle production whereas the IFN-sensitive, green fluorescent protein-expressing vesicular stomatitis virus (VSV-GFP) infection was controlled. Further evaluation of the other IFN-α/β subtypes versus KFDV infection indicated that single treatments of either IFN-αWA and IFN-αΚ appeared to be more effective than IFN-α2a at reducing KFDV titres. Concentration-dependent analysis of these IFN-α/β subtypes revealed that regardless of subtype, low concentrations of IFN were able to limit cytopathic effects (CPE), while significantly higher concentrations were needed for inhibition of virion release. Furthermore, expression of the KFDV NS5 in cell culture before IFN addition enabled VSV-GFP to overcome the effects of IFN-α/β signalling, producing a robust infection.

**Conclusions/Significance:**

Treatment of cell culture with IFN does not appear to be suitable for KFDV eradication and the assay used for such studies should be carefully considered. Further, it appears that the NS5 protein is sufficient to permit KFDV to bypass the antiviral properties of IFN. We suggest that other prophylactic therapeutics should be evaluated in place of IFN for treatment of individuals with KFDV disease.

## Author Summary

Since 1957 Kyasanur Forest disease virus (KFDV) has caused seasonal infections and periodic outbreaks in south-west India. It is estimated that nearly 500 people acquire KFDV annually and 3–5% of those infected succumb to the disease. The vaccine strategy is complicated by the lack of coverage, compliance and efficacy, highlighted by the fact that less than half of the target population received the recommended three dose-regimen. Besides the prevention of tick bites and vaccination, there are no approved antivirals for KFDV infection. Based on these observations, the commonly-used-IFN-α2a was assessed and was not capable of limiting KFDV virus titres. Further characterization of the other IFN-α/β subtypes used at different concentrations revealed that KFDV replication was insensitive to all subtypes, even though signs of cellular damage were reduced. Thus, infectious titre, rather than monolayer staining or cytopathic effect (CPE) monitoring, is more reliable for IFN analyses. The capability of KFDV to overcome the antiviral properties of IFN was attributed to the NS5 protein. Thus, other treatment options need to be evaluated for patients suffering with Kyasanur Forest disease.

## Introduction

Kyasanur Forest disease virus (KFDV) is a tick-borne flavivirus that was identified in 1957 following a monkey epizootic and a coinciding human outbreak in south-west India [1]. KFDV cases previously were localized within the Shimoga district of Karnataka; however KFDV has been recently discovered in the neighboring states of Kerala, Tamil Nadu, Goa and Maharashtra [2–5] and, possibly China in 1989 [6] increasing the potential public health risk associated with this pathogen. A vaccine for KFDV is available for those living in affected areas and those living within a 5 kilometer radius of a positive case from either humans, monkeys or tick pools [7], but there has been issues with implementation and efficacy. The most troubling aspect of vaccine use is that less than half of the target population actually receive the full three-dose regimen that is required for protection [8,9]. With the annual number of cases ranging from 400–500 and an associated fatality rate of 3–5% [10], there is a need for alternative therapeutic options, besides the current vaccine and tick bite prevention measures.

KFDV is a member of the tick-borne encephalitis serocomplex which includes: tick-borne encephalitis, the former Russian spring-summer encephalitis, Omsk hemorrhagic fever, Powassan, Langat and Louping-Ill viruses [11]. A variant of KFDV, Alkhumra hemorrhagic fever virus located in Saudi Arabia [12] and in Egypt [13–15], also is part of this complex [16]. The single-stranded positive-polarity RNA genome of KFDV is 10, 774 bases in length and encodes a single polyprotein: C-prM-E-NS1-NS2A-NS2B-NS3-NS4A-NS4B-NS5 [17]. KFDV, Alkhumra hemorrhagic fever virus and Omsk hemorrhagic fever virus are unique to this complex as they primarily cause hemorrhagic fever manifestations with neurological involvement [18].

Interferon (IFN) was first described for its ability to interfere with virus infection in 1957 by Isaacs and Lindenmann [19,20]. In response to viral infection, IFN is released from infected cells to surrounding uninfected cells. Upon binding to its receptor and subsequent activation of the Jak/STAT signaling cascade, the cellular “antiviral state” is established. The antiviral state allows naïve cells to become resistant to viral infection via expression of IFN-stimulated genes (ISG) that have many roles in protecting the host from infection [21–23]. KFDV, like many other flaviviruses including dengue, yellow fever, Langat, West Nile, tick-borne encephalitis and Japanese encephalitis viruses [24–27] employs the NS5 protein to prevent antiviral state establishment and compromised IFN signalling [28], whereas NS4B-2k has been also implicated for dengue, West Nile and yellow fever viruses [26,29,30]. The ramifications of a compromised Jak/STAT pathway were highlighted in mice lacking the IFN-α/β receptor. Infection of these mice with West Nile [31], dengue [32] and Langat viruses [33] led to increased virus burden, earlier onset of clinical signs and higher mortality when compared to control mock-infected type mice.

Of the type 1 IFNs (14 subtypes of α and one of β), only IFN-α2a and α2b are FDA-approved for clinical treatment in humans [22]. Case reports indicate a relatively narrow window for post-exposure treatment against other flaviviruses such as West Nile, Saint Louis encephalitis and Japanese encephalitis viruses with either IFN-α2a or 2b, especially in the immune-compromised or during the severe stages of disease [34–37]. In tissue culture, both IFN-α2a and α2b are potent at reducing the replication of dengue virus 2 [38,39], West Nile [31,40,41], Langat [25] and other flaviviruses [42]. Interestingly, strain-specific responses were observed between dengue virus 1, 3 and 4, yellow fever virus (vaccine strains FNV and 17D) [42] and Japanese encephalitis virus (strains Vip, KE-093, KE-094 and KE-095) [43]. Similarly, the antiviral activities of other IFN-α/β subtypes differ dramatically for vesicular stomatitis virus (VSV), human immunodeficiency virus [44] and human rhinoviruses [45]. To our knowledge, with the exception of IFN-α2a, the other IFN-α/β subtypes have not been evaluated against KFDV.

Current treatment options for KFDV are limited to supportive care, because no therapeutic drugs have been clinically approved. Using VSV as an IFN-sensitive virus control for comparison, the antiviral efficacy of the IFN-α/β subtypes was assessed against KFDV infection in cell culture. We conclude that IFN-α2a was unable to eradicate KFDV from cell culture, but appeared to offer protection form cytopathic effects (CPE). Although some IFN subtypes at higher doses did restrict KFDV replication, albeit marginally, further scrutiny could not demonstrate a strong dose-dependent response in terms of virion production Thus, it is not appropriate to use protection from CPE as the sole determinant of potency. In conclusion, KFDV is not sensitive to the antiviral effects of IFN and despite the lack of a concise mechanism for IFN resistance, it is clear that the NS5 protein is a major contributor.

## Methods

### Cells, Viruses and Interferon

#### Cells

For all experiments, human lung carcinoma (A549 CCL-185) cells (ATCC, Burlington, Ontario, Canada) and African green monkey kidney (VeroE6 CRL-1586) cells (ATCC) were propagated in Dulbecco’s modified eagle medium (DMEM) (HyClone, Ottawa, Ontario, Canada) growth medium supplemented with 10% serum (Gibco, Burlington, Ontario, Canada) and 1% antibiotics (Penicillin-Streptomycin) (Gibco). Baby hamster kidney (BHK-21 CCL-10) cells (ATCC) were grown in growth medium, Eagle’s minimum essential medium (EMEM) (HyClone) with 10% serum and 1% antibiotics. Alternatively, during infections the serum content in each media type was reduced to 2% and the resulting formulation is referred to as virus maintenance medium. All cell lines were incubated during propagation or infection at 37°C/5% CO_2_, unless stated otherwise.

#### Viruses

KFDV (P9605 strain, GenBank accession number: HM055369) stocks were propagated in VeroE6 cells in Containment Level 4 (CL-4) laboratory of the National Microbiology Laboratory (NML) at the Canadian Science Centre for Human and Animal Health (CSCHAH) in Winnipeg, Canada. Supernatants were harvested at 96 hours post-infection (hpi). Stocks of vesicular stomatitis virus expressing green fluorescent protein (VSV-GFP) were propagated in VeroE6 cells and harvested at 72 hpi.

#### Interferon

Human type 1 IFN (α-1 through 14, β-1) and Universal IFN (recombinant αA/D) were obtained from PBL Assay Science (Burlington, On., Canada), diluted to 50, 000 U/ml and stocks were stored at -80°C.

### Virus Clearance by IFN-α2a Treatment

A549 and BHK cells were seeded in 6-well plates for 80–90% confluency at time of infection. The cells were infected with 11 TCID_50_ units (equivalent to a multiplicity of infection (MOI) of 0.00001) of KFDV or VSV-GFP for 1 hour, then virus maintenance medium supplemented with a final concentration of 2, 000 U/ml of IFN-α2a was added and incubated for 96 hours (KFDV) and 48 hours (VSV-GFP); this was designated as passage 0. Controls wells were treated similarly without the addition of IFN. Supernatants were harvested and stored at -80°C for titration. The infected-cells were washed with PBS, passaged by use of trypsin (HyClone) and the cells were split (1:3) into two new 6 well plate wells. These passage 1 samples were treated again with 2, 000 U/mL IFN-α2a or were left untreated and re-incubated for 72 hours (KFDV) and 48 hours (VSV-GFP). The procedure was repeated from passage 1 to passage 2. Pictures from virus-infected cells were captured using an EVOS microscope for KFDV samples under CL-4 conditions and with an Axiovert 40 CFL microscope (Carl Zeiss, Toronto, Ontario, Canada) for VSV-GFP samples under CL-2 conditions.

### Screening of IFN α/β Subtypes against KFDV

A549 cells were prepared in 24-well plates with DMEM growth medium for 80–90% confluency at time of infection. In the pre-treatment scenario, the cells were treated with or without 1, 000 U/ml of the selected IFN-α/β subtypes was added and incubated 24 hours before virus infection. Then the cells were infected with KFDV at a MOI of 1 for 1 hour, inoculum was removed, cell monolayers were washed and virus maintenance medium was added. In the post-treatment experiments, KFDV at a MOI of 1 was adsorbed for 1 hour, inoculum was removed, monolayers were washed, and then fresh virus maintenance medium supplemented with or without 1, 000 U/ml of the selected IFN α/β subtypes. Supernatants from three-independent biological replicates were harvested and stored at -80°C for virus quantification after an incubation period of 72 hours. Statistical significance was determined using One-way ANOVA analysis followed by Tukey’s post-test.

### Dose-Dependent Antiviral Activity of IFN-αWA, and IFN-α2a against KFDV and VSV-GFP

#### Inhibitory concentrations and titre reduction

A549 and BHK-21 cell cultures were prepared in 96-well plates for 80–90% confluence. Cells were infected with 50 μl/well of 11 TCID_50_ units of KFDV or VSV-GFP (equivalent to a MOI of 0.0003) in three independent, triplicate replicates. After an adsorption period of 1 hour, 150 μl of fresh virus maintenance medium was added with IFN-α2a or αWA prepared following a two-fold dilution scheme (4, 000.0 U/ml—3.9 U/ml). Control samples were mock-treated and infected and, mock-treated and uninfected. When mock-treated infected control reached 90–100% CPE at 96 hpi (KFDV) or 48 hpi (VSV-GFP), supernatants from 2, 000.0, 500.0, 62.5, 7.8 U/mL and mock-treated controls were harvested and stored at -80°C for titration and the monolayers were fixed for IC_50_ and IC_90_ determination. Cell monolayers were treated with 10% phosphate-buffered formalin, washed with PBS and stained with 0.5% (wt/vol) crystal violet (Fisher Scientific, Ottawa, Ontario, Canada) dissolved in a solution of 70% methanol/30% PBS. After incubation for 30 minutes at room temperature, the excess dye was removed with tap water. Once air-dried, crystal violet dye was eluted in 100 μl of 95% ethanol and scanned with a MultiSkan Accent microplate reader at 570 nm (Thermo Scientific, Mississauga, Ontario, Canada). The inhibitory concentration is the amount of IFN (αWA or α2a) needed to protect 50% (IC_50_) and 90% (IC_90_) of cells from virus-induced CPE (KFDV and VSV-GFP). These values were compared to the un-infected controls which represented cells without CPE (100%) and to the infected controls defined as full CPE (0% protection). Values were calculated with curve fitting using GraphPad software (Prism 5). For the reduction of virus titres, un-infected controls represented a lack of virus production and full virus infection titres were defined by the infected/mock-treated controls.

#### Virus quantification

BHK-21 cells were seeded into 96-well plates for 80–90% confluency. Virus-containing supernatants (KFDV or VSV-GFP) were serially diluted 10-fold. Fifty μl from each dilution was added to triplicate wells and incubated for 1 hour. Then, 150 μl of virus maintenance medium was added and re-incubated for CPE development that was evaluated 5 days post-infection for KFDV or 72 hpi for VSV-GFP. The 50% tissue culture infectious (TCID_50_/mL) dose was calculated following the Reed and Muench formula [46].

#### Cellular cytotoxicity

A549 and BHK-21 cells cell cultures were prepared in 96-well plates for 80–90% confluency and IFN-αWA and α2a were added following a two-fold dilution scheme from 16, 000.0–3.9 U/mL. For control purposes, low toxicity (mock-treated) and high toxicity (10% Triton X-100) (Sigma, Oakville, Ontario, Canada) were included for the analysis. After incubation for 96 hours, monolayers were prepared and subjected to crystal violet staining, as described above. The concentration of IFN that caused 50% cell death was defined as the cytotoxicity concentration (CC_50_). Values were calculated with curve fitting using GraphPad software (Prism 5).

### IFN α/β Subtypes Antagonism Assays

#### Cloning

KFDV NS5, NS4B-2k and Ebola virus VP24 genes were cloned into the pCAGGS mammalian expression-vector under chicken beta-actin promoter control, as described previously [28].

#### VSV-GFP infection assays

VeroE6 cells were transfected using Fugene 6 (Promega, Madison, Wi., USA) with pCAGGS expressing KFDV NS5, NS4B-2k, VP24 proteins or mock transfected with empty pCAGGS vector following the manufacturer’s recommendations. Cells were then treated, post-transfection with virus maintenance medium containing 1, 000 U/mL of each IFN-α/β subtypes or Universal IFN for 24 hours. The supernatants were removed and cells were infected with VSV-GFP (MOI of 2) in 500 μl of virus maintenance medium for 1 hour at 37°C/5% CO_2_. Virus inoculum was removed and monolayers were washed once with virus maintenance medium and fresh maintenance medium was added. After an incubation period of 24 hours, pictures were taken with light and fluorescent microscopy with an Axiovert 40 CFL microscope (Carl Zeiss) and supernatants were harvested and stored at -80°C until quantification.

## Results

### IFN-α2a Treatment Does Not Clear KFDV Infection *In Vitro*

Using virus clearance assays that involved successive passaging of infected cell cultures along with the re-application of antivirals [47,48], we investigated the efficacy of IFN-α2a against KFDV and for control purposes, against VSV-GFP. To address this, both A549 and BHK-21 cells were infected with either KFDV or VSV-GFP at 11 TCID_50_ units (MOI of 0.0001). After infection the cells were treated with 2, 000 U/mL of IFN-α2a at 1 hpi (passage 0). Virus supernatants were harvested for titration, and passage 0 infected-cells were subcultured into two wells and IFN was either added or withheld (passage 1) and this procedure was repeated for passage 2.

**Fig 1:**
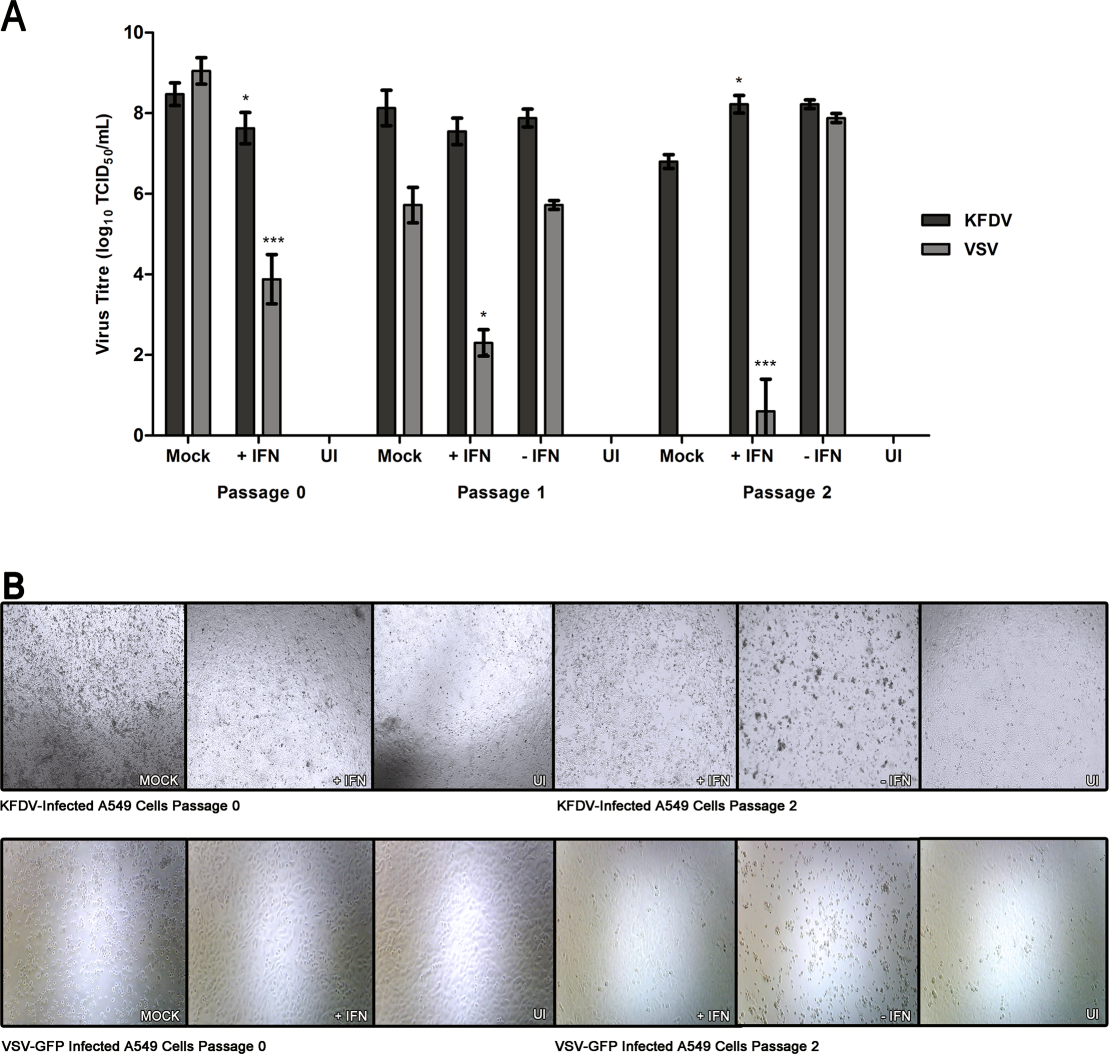
IFN-α2a does not clear KFDV infection in A549 cells. A549 cells were infected with a 11 TCID_50_ units (MOI of 0.00001) of the indicated virus, and either treated or mock-treated with 2, 000 U/mL of IFN-α2a (designated as P0). Monolayers were passaged when untreated controls reached CPE of 90%, 96 and 48 hpi for KFDV and VSV-GFP, respectively and 2, 000 U/mL of IFN-α2a was either added or omitted (P1) and after 72 hpi for KFDV and 48 hpi for VSV-GFP. This procedure was repeated again for passage 2 (P2). (A) Before each passage, supernatants were harvested for titration by TCID_50_ assay determination on BHK-21 cells. The averages and standard deviation from three biological replicates are shown graphically and expressed in log_10_ scale TCID_50_/mL. Statistical significance is denoted as * P < 0.1, ** P < 0.05, *** P < 0.01. (B) Cell monolayers were visualized with light microscopy prior to passaging. Mock, non-IFN treated/infected controls. UI, Un-infected controls.

**Fig 2:**
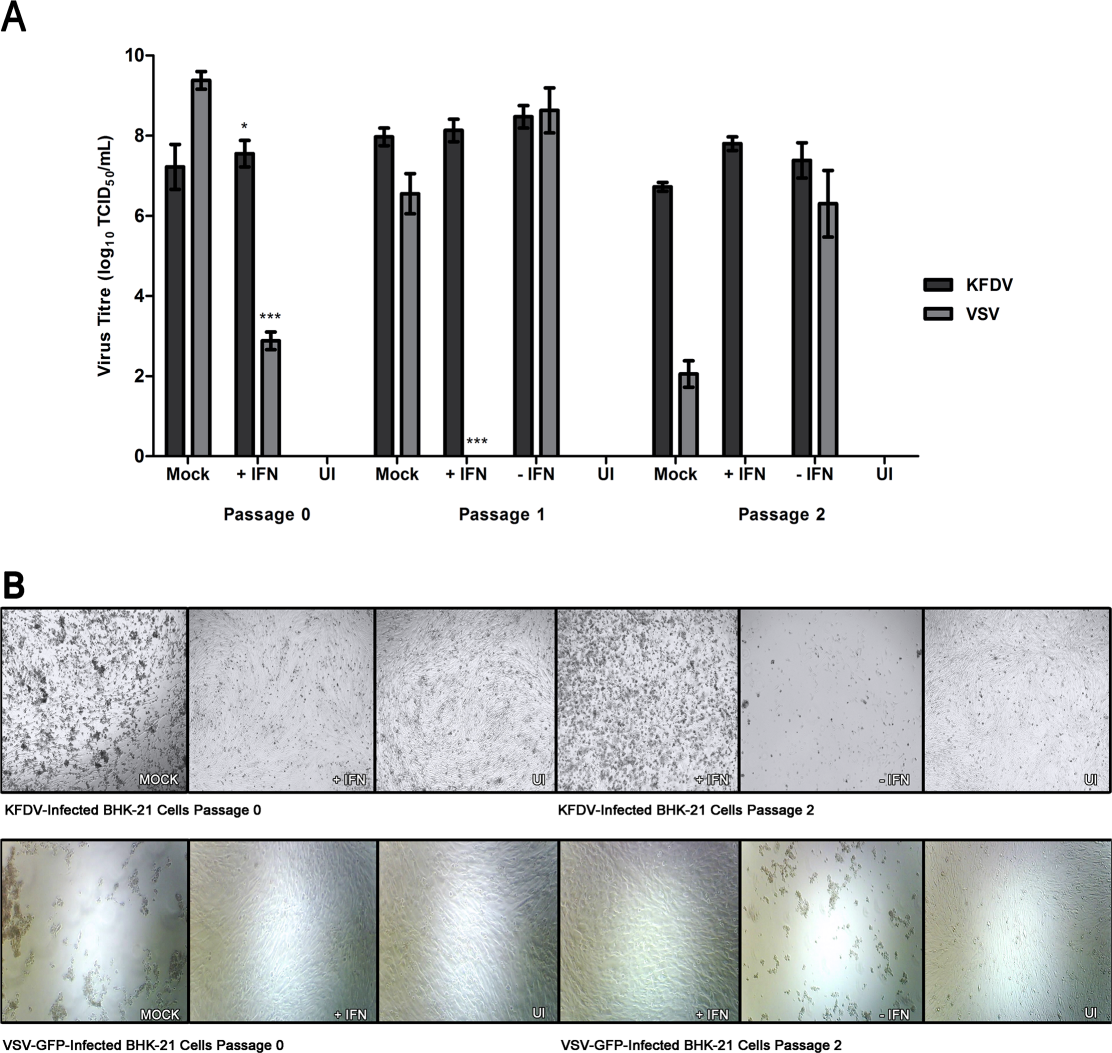
IFN-α2a does not clear KFDV infection in BHK-21 cells. BHK-21 cells were infected with a 11 TCID_50_ units (MOI of 0.00001) of the indicated virus, and either treated or mock-treated with 2, 000 U/mL of IFN-α2a (designated as P0). Monolayers were passaged when untreated controls reached CPE of 90%, 96 and 48 hpi for KFDV and VSV-GFP, respectively and 2, 000 U/mL of IFN-α2a was either added or omitted (P1) and after 72 hpi for KFDV and 48 hpi for VSV-GFP. This procedure was repeated again for passage 2 (P2). (A) Before each passage, supernatants were harvested for titration by TCID_50_ assay determination on BHK-21 cells. The averages and standard deviation from three biological replicates are shown graphically and expressed in log_10_ scale TCID_50_/mL. Statistical significance is denoted as * P < 0.1, ** P < 0.05, *** P < 0.01. (B) Cell monolayers were visualized with light microscopy prior to passaging Cell monolayers were photographed prior to passaging. Mock, non-IFN treated/infected controls. UI, Un-infected controls.

Initially the passage 0, IFN-treated cells showed a reduction in KFDV titre from the mock-treated controls of 10^0.8^ TCID_50_/mL (p< 0.1) and 10^0.4^ TCID_50_/mL (p< 0.1) in both A549 and BHK-21 cells respectively. However, decreases were more striking for the IFN-sensitive VSV-GFP as reductions of 10^5.2^ TCID_50_/mL (p< 0.01) and 10^6.5^ TCID_50_/mL (p< 0.01) were observed in A549 and BHK-21 cells respectively (Fig 1A and Fig 2A).

When comparing the IFN-treated cells from passage 0 to passage 1 and passage 1 to passage 2, only VSV-GFP showed marked differences in titres. There were no apparent decreases in KFDV titres produced from IFN-treated A549 cells (passage 1: decrease of 10^0.1^ TCID_50_/mL (not significant) from passage 0, and for passage 2: increase of 10^0.7^ TCID_50_/mL (p< 0.1) from passage 1)(Fig 1A) and from IFN-treated BHK-21 cells (passage 1: increase of 10^0.6^ TCID_50_/mL (not significant) from passage 0, and passage 2: decrease of 10^0.3^ TCID_50_/mL (not significant) from passage 2) (Fig 2A). The results from KFDV contrast that of VSV-GFP in which sharp declines in titres were observed in A549 cells (passage 1: decrease of 10^1.6^ TCID_50_/mL (p< 0.1) from passage 0, and a reduction of 10^1.7^ TCID_50_/mL (p< 0.01) from passage 1)(Fig 1A) and BHK-21 cells (passage 1: a decrease of 10^2.9^ TCID_50_/mL (p< 0.01), and passage 2: no virus detected) cells (Fig 2A).

As anticipated, when IFN-treated cells from either passage 0 or passage 1 were subcultured and not further treated with IFN, virus titres and CPE for both KFDV and VSV-GFP were characteristic of the mock-treated passage 0 controls. Despite the apparent inability of IFN to significantly impact KFDV propagation, the appearance of CPE was notably reduced compared to mock-treated in both cell types tested (Fig 1B and Fig 2B). Even with the lack of visible CPE in the IFN-treated cells, KFDV could not be eliminated from infected cell culture by treatment with IFN-α2a.

### Preliminary Screening of IFN-α/β Subtypes with Time of Addition against KFDV

Previous studies evaluating IFN treatment against flaviviruses in cell culture have revealed that IFN is more effective when added to cells before virus infection and becomes less effective over time once infection has been established [25,31,39,41]. To further expand our understanding of the efficacy of IFN treatment against KFDV infection, we evaluated the antiviral activity of the IFN-α/β subtypes in both pre-infection and post-infection time frames.

**Fig 3:**
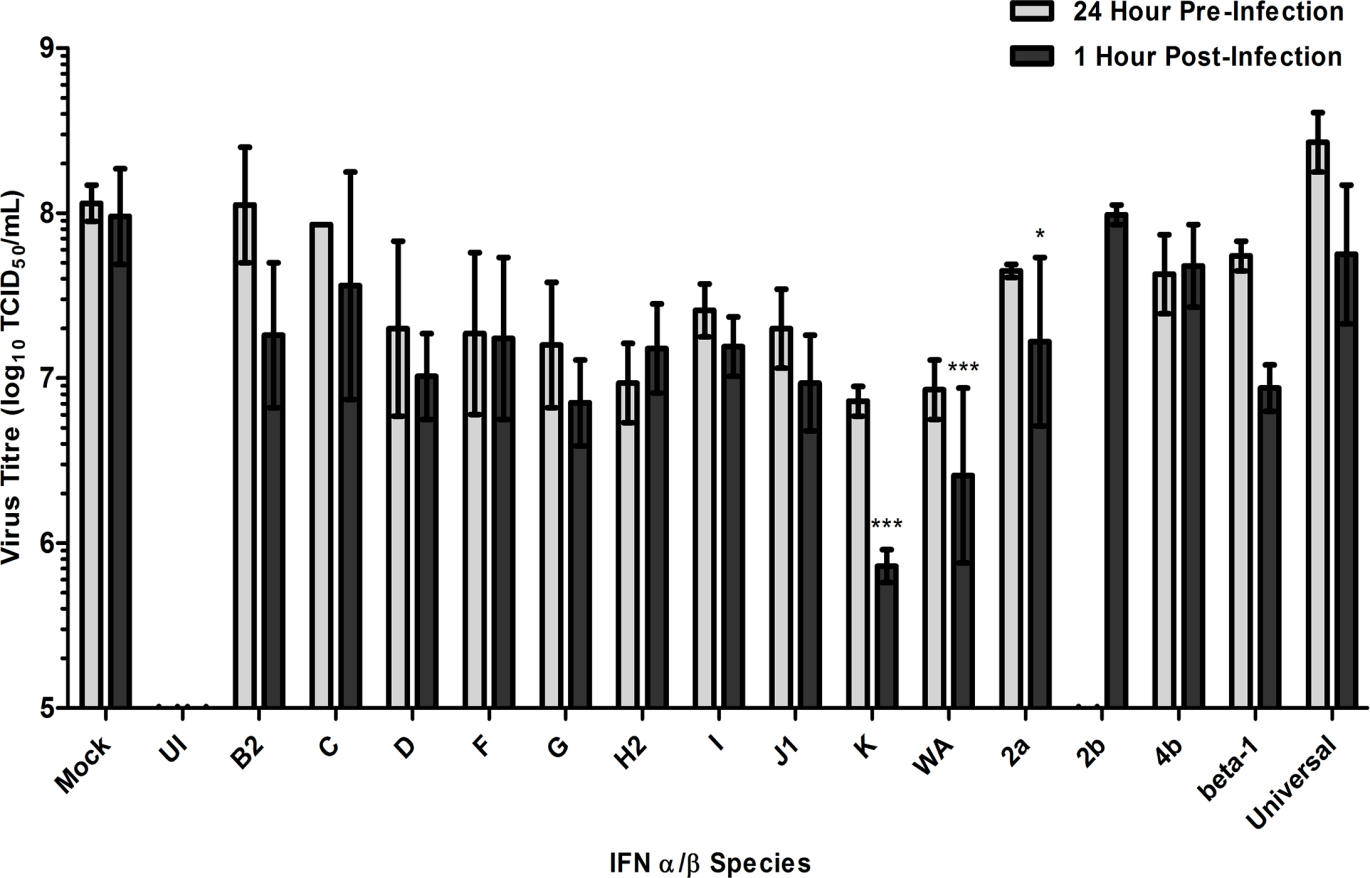
Screening interferon-α/β subtypes against KFDV. Cultures of A549 cells were pre-treated (grey bars) 24 hours prior to infection or post-treated (black bars) 1 hour after infection with 1, 000 U/mL of IFN-α (B2, C, D, F, G, H2, I, J1, K, WA, 2a, 2b or 4b), IFN-β (beta-1) and a recombinant IFN-α (Universal) subtypes and infected with KFDV at a MOI of 1. Supernatants were harvested for each treatment after 72 hours of incubation and quantified (expressed in log_10_ scale TCID_50_/mL) on BHK-21 (ATCC) when the mock-treated control cells displayed CPE near 100%. Pre-infection treatment experiments were assayed in two biological replicates and post-infection treatment experiments were assayed in three biological replicates; the resulting averages and standard deviations are presented. Mock, Mock-treated with IFN. UI, Un-infected control. IFN-α2b was excluded from 24-hour pre-infection treatment. * Significant compared to mock-treated samples (P < 0.1). *** Significant compared to mock-treated samples (P < 0.01).

A549 cells were infected with KFDV (MOI of 1) and treated with 1, 000 U/mL of each IFN-α/β, which was added either 24 hours before or 1 hour after infection. When CPE reached 90–100% in mock-treated controls, supernatants were harvested and titrated. Regardless of time of addition, both IFN-αK and IFN-αWA were more effective than IFN-α2a at reducing KFDV titres (Fig 3). When compared to the mock-treated controls, fold reductions in pre-infection and post-infection time frames were 16-fold (IFN-αK), 14-fold (IFN-αWA) and 3-fold (IFN-α2a) (Fig 3, grey bars) and, 132-fold (IFN-αK p<0.01), 37-fold (IFN-αWA p<0.01) and 6-fold (IFN-α2a p<0.1) (Fig 3, black bars), respectively.

These data suggest that IFN-αK and IFN-αWA may induce responses that are more effective than those produced by IFN-α2a for restricting KFDV infection regardless of the time of treatment relative to infection.

### Dose-Dependent Antiviral Activity of IFN-αK, IFN-αWA and IFN-α2a

The two candidate IFN subtypes, IFN-αK and IFN-αWA were observed to be more effective than IFN-α2a at repressing KFDV replication at a concentration of 1, 000 U/mL. To further explore this finding, we sought to determine if the potency differences of IFN-αWA compared to IFN-α2a can be maintained over a range of concentrations. This was tested by analyzing the ability of each IFN subtypes to protect A549 and BHK-21 cells from the cytopathology of KFDV (11 TCID_50_ units equal to MOI of 0.0003) and a IFN-sensitive control virus (VSV-GFP) (11 TCID_50_ units equal to MOI of 0.0003) following a two-fold dilution scheme for each IFN. The traditional approach to defining IFN potency was utilized; these assays involve the use of multiple cell types including A549 cells, treated with IFN and challenged with a strong cytolytic virus like VSV, then analyzed empirically with monolayer staining/spectroscopy [21,49–51]. As an additional measure, IFN strength was assessed by infectious virion production determined by TCID_50_ titration from select IFN concentrations.

For the cytopathology assessment, upper and lower parameters were defined as either full CPE (0% cell protection) from mock-treated samples and absence of CPE (100% cell protection) from un-infected samples, thereby allowing IC_50_ and IC_90_ to be determined for each IFN. Initially both IFN subtypes appeared equally as potent, as the IC_50_ values for IFN-αWA and IFN-α2a were comparable against KFDV (5.2 and 7.4 U/mL) and VSV-GFP (5.1 and 5.6 U/mL) in A549 cells (Table 1). However, the same trend was not observed in BHK-21 cells with IC_50_ values of 23.3 (IFN-αWA) and 6.9 U/mL (IFN-α2a) for KFDV. An IC_50_ of 988.1 U/mL was determined for IFN-α2a against VSV-GFP and a value could not be determined (ND) for IFN-αWA. This may have been because the amount of IFN required to reach the IC_50_ was greater than the highest concentration tested and therefore could not be determined using the curve fitting software (Table 1). IC_90_ values obtained from KFDV-infected A549 cells that were treated with IFN-αWA (406.8 U/mL) and IFN-α2a (125.4 U/mL) were significantly higher than VSV-GFP-infected A549 cells that were treated with IFN-αWA (48.5 U/mL) and IFN-α2a (50.0 U/mL) (Table 1). A similar trend was observed for KFDV-infected BHK-21 cells. The 90% protection from IFN-αWA (23, 711.0 U/mL) was extrapolated by curve fitting software as it was much greater than the highest amount tested (16, 000.0 U/mL). This predicted value was significantly higher than that of IFN-α2a (2, 048.0 U/mL). With respect to VSV-GFP-infected BHK-21 cells that were treated with IFN, the IC_90_ was determined to be 3, 808.0 U/mL for IFN-αWA and 56.2 U/mL for IFN-α2a (Table 1). IFN-induced cytotoxicity (CC_50_) was not determined as a concentration of 16, 000.0 U/mL, an amount that was three times larger than the highest concentration used for experiments led to cell death in A549 (25% cytotoxicity) and BHK-21 (11% cytotoxicity) cells. For control parameters, un-treated (0% cytotoxicity) and 10% Triton X-100-treated (100% cytotoxicity) samples defined the upper and lower limits of CC_50_ (Table 1). While it was apparent that high concentrations of both IFN-αWA and IFN-α2a are required to prevent KFDV-induced CPE, the experiments utilizing BHK-21 cells highlight the problems of obtaining qualitative data for IFN-protection via CPE, especially against VSV-GFP.

**Table 1:** Antiviral activity of interferon-α/β against the cytopathology of KFDV and VSV-GFP.

Interferon (IFN) Species	KFDV IC_50_ (U/mL)	VSV-GFP IC_50_ U/mL)	KFDV IC_90_ (U/mL)	VSV-GFP IC_90_ (U/mL)	CC_50_ (U/mL)
A549^a^					
WA	5.2	5.1	406.8	48.5	> 16, 000.0
2a	7.4	5.6	125.4	50.0	
K	18.8	ND^c^	108.0	ND^c^	
BHK-21^b^					
WA	23.3	ND^c^	23, 711.0	3, 808.0	> 16, 000.0
2a	6.9	988.1	2, 048.0	56.2	

^a^ IC_50_, CC_50_ and IC_90_ values from three technical replicates of each of three biological replicates in A549 cells.

^b^ IC_50_, CC_50_ and IC_90_ values from three technical replicates of each of one biological replicates in BHK-21 cells.

^c^ ND = not determined.

To further define IFN potency with respect to the variation of IC values and dose-dependence, virus titres from a range of IFN concentrations (2, 000.0, 500.0, 62.5 and 7.8 U/mL) selected from the two-fold IFN dilution experiments in both A549 and BHK-21 cells were compared to un-treated controls. This was compared as titre reductions for both KFDV and VSV-GFP (control virus). For the highest (2, 000.0 U/mL) and lowest (7.8 U/mL) concentrations assessed for KFDV-infected A549 cells, titre reductions of 10^0.7^ and 10^0.5^ TCID_50_/mL (IFN-αWA) and, 10^0.5^ and 10^0.4^ TCID_50_/mL (IFN-α2a), respectively, were measured. In BHK-21 cells infected with KFDV, titre reductions were not observed for 2, 000.0 and 7.8 U/mL of IFN-αWA; however, reductions of 10^0.9^ and 10^0.1^ TCID_50_/mL were observed for IFN-α2a. By contrast in VSV-GFP, titre reductions were more pronounced in the A549 cells treated with both 2, 000.0 U/mL (10^7.9^ TCID_50_/mL for IFN-αWA and 10^6.9^ TCID^50^/mL for IFN-α2a) and 7.8 U/mL (10^3.6^ TCID_50_/mL for IFN-αWA and 10^3.5^ TCID_50_/mL for IFN-α2a). And for BHK-21 cells treated with 2, 000.0 U/mL (10^1.8^ TCID_50_/mL for IFN-αWA and 10^4.4^ TCID_50_/mL for IFN-α2a) and 7.8 U/mL (10^0.3^ TCID_50_/mL for IFN-αWA and 10^1.5^ TCID_50_/mL for IFN-α2a) (Table 2).

**Table 2:** Antiviral activity of interferon-α/β against KFDV and VSV-GFP virion production.

Interferon (IFN) Species	Concentration (U/mL)	Virus Titre Reduction From Mock-Treated^c^	Virus Titre Reduction From Mock-Treated^c^
A549		KFDV	VSV-GFP
2a	2, 000.0	0.5	6.9
	500.0	0.7	7.5
	62.5	0.7	4.7
	7.8	0.4	3.5
WA	2, 000.0	0.7	7.9
	500.0	0.6	7.0
	62.5	1.1	6.2
	7.8	0.5	3.6
K	2, 000.0	0.6	ND
	7.8	0.2	ND
Mock-Treated^a^	0.0	0.0	0.0
BHK-21		KFDV	VSV-GFP
2a	2, 000.0	0.9	4.4
	500.0	0.5	1.2
	62.5	0.1	0.7
	7.8	0.1	1.5
WA	2, 000.0	0.0	1.8
	500.0	0.1	-0.4
	62.5	0.2	0.1
	7.8	0.0	0.3
Mock-Treated^b^	0.0	0.0	0.0

^a^ Titre reduction of mock-treated samples were calculated from original titres in A549 cells of 8.2 and 9.5 log_10_ TCID_50_/mL for KFDV and VSV-GFP, respectively.

^b^ Titre reduction of mock-treated samples were calculated from original titres in BHK-21 cells of 7.2 and 9.1 log_10_ TCID_50_/mL for KFDV and VSV-GFP, respectively.

^c^ Titre reduction expressed in log_10_ scale TCID_50_/mL.

When comparing VSV-GFP to KFDV, it is clear that KFDV is not sensitive to the effects of IFN. While the traditional monolayer staining procedure demonstrated a dose-dependent relationship between CPE and IFN concentration, the assay showed variability from experiment to experiment, especially in BHK-21 cells. The measurement of virus titre proved more reliable as the true IFN-insensitive nature of KFDV was revealed.

### Assessing the Potential of KFDV NS5 to Subvert IFN Signalling

Similar to other flaviviruses, our previous work had revealed that the NS5 protein and not the NS4B-2k protein of KFDV could inhibit IFN signaling by interrupting the Jak/STAT pathway [28]. We characterized this further in hopes of determining if the failure of the IFN-α/β subtypes to restrain KFDV replication was due to the effects of NS5.

**Fig 4:**
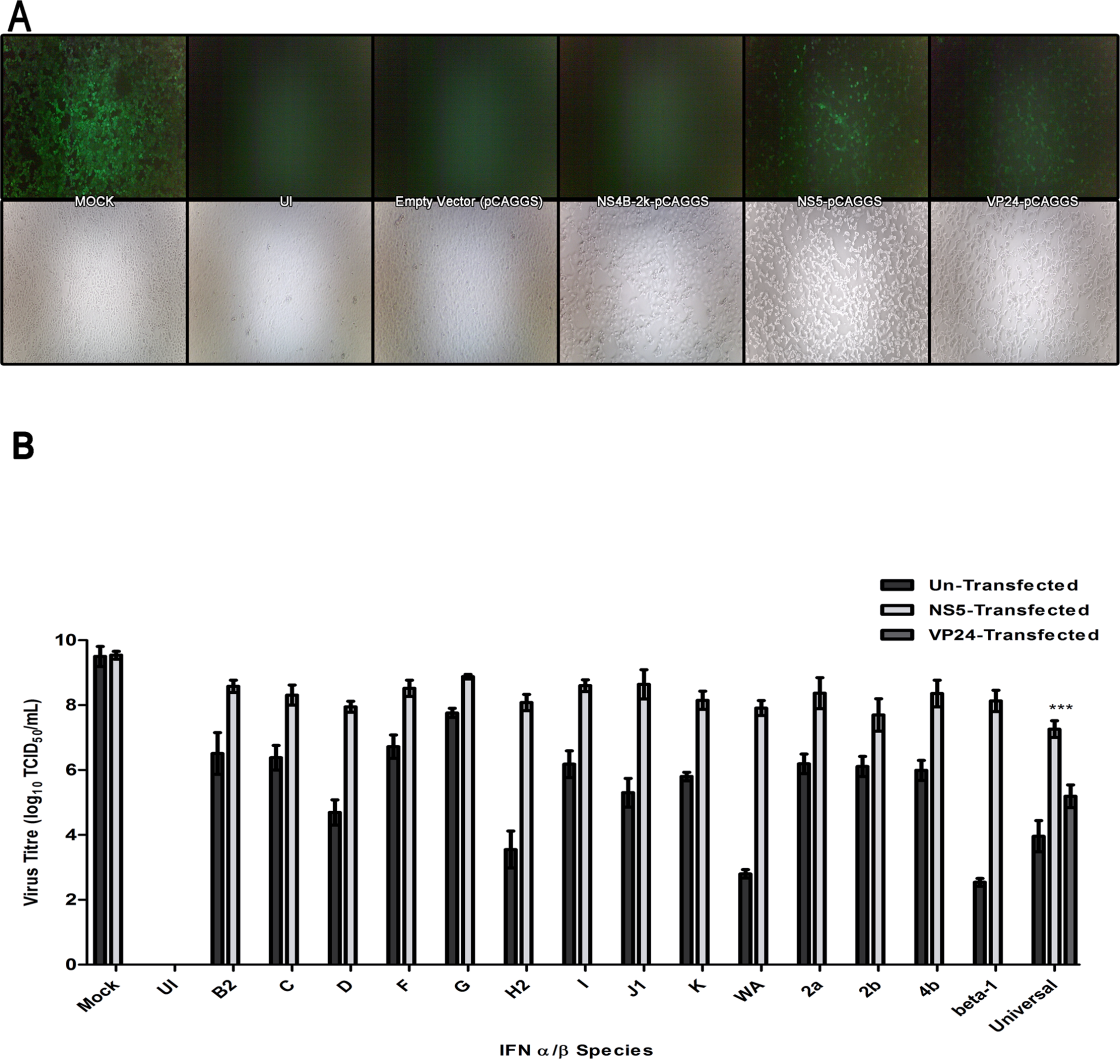
KFDV NS5 impedes the cellular antiviral state. **(A)** VeroE6 (ATCC) cells were transfected with plasmid encoding KFDV NS proteins and Ebola virus VP24 and treated with 1, 000 U/mL of Universal IFN, 24 hpi. After a 24-hour incubation period, cells were infected with VSV-GFP (MOI of 2) and, pictures were taken with fluorescent (top panel) and light (bottom panel) microscopy 24 hours later. **(B)** VeroE6 (ATCC) cells were transfected with KFDV NS5-pCAGGS and treated with 1, 000 U/mL of commercially available type I IFNs, 24 hpi. After a 24-hour incubation period, cells were infected with VSV-GFP (MOI of 2) and, 24 hours later, the virus-containing supernatants were harvested for virus quantification. Dark grey bars indicate experiments in which cells were un-transfected. Light grey bars indicate NS5-expressing cells. Mock, no IFN treatment of cells lacking NS5 expression (dark gray bar) and with NS5 expression (light gray bar); UI represents uninfected/un-treated cells. Universal IFN controls included VP24-pCAGGS as anti-IFN control. The graph represents the log_10_ scale TCID_50_/mL averages and standard deviations from three biological repetitions. ***, Significant difference of NS5-expressing cells compared to VP24-expressing cells (P < 0.01).

VeroE6 cells were transfected with plasmids expressing the KFDV: NS5 protein and NS4B-2k proteins which previously have been shown to have anti-IFN and lacking anti-IFN activity, respectively [28] and, for control purposes the Ebola virus VP24 protein which is an IFN-signaling repressor [52]. The cells were then treated with 1, 000 U/mL of Universal IFN with exception of the mock-treated control, before being infected with VSV-GFP (MOI of 2). The mock-treated controls demonstrated extensive CPE and GFP expression indicating an un-inhibited infection (Fig 4A). As anticipated IFN prevented VSV-GFP propagation in NS4B-2k and empty-expression vector controls as indicated by CPE and GFP expression and these were comparable to those seen in uninfected cells. VSV-GFP was only able to overcome the effects of IFN when NS5 was present and this was similar to the VP24 control (Fig 4A).

With the KFDV NS5 established as a primary antagonist against Universal IFN activity, the assay was extended to compare the ability of NS5 to circumvent the other IFN-α/β subtypes measured by VSV-GFP infectious particle production. VeroE6 cells were either transfected with plasmids expressing NS5, VP24, or mock-transfected, then treated with 1, 000 U/mL of each IFN-α/β subtypes, with exception of a mock-treated control and, finally infected with VSV-GFP (MOI of 2). When mock-treated controls reached 90–100% CPE, virus-containing supernatants were harvested and titrated. The mock-treated cells were susceptible to VSV-GFP and NS5 expression did not negatively-impact infection as 10^9.5^ TCID_50_/mL was detected under both circumstances. All of the IFN-α/β subtypes limited VSV-GFP replication (dark grey bars); however, the expression of NS5 reversed the antiviral effects of all IFN subtypes (light grey bars). Therefore, NS5 was able to recover VSV-GFP and permitted virus titres to increase by (reported from lowest to highest): 10^1.1^ for IFN-αG to 10^5.1^ for IFN-αWA and, 10^5.6^ for IFN- β (Fig 4B). Furthermore, NS5 appeared to be a stronger repressor of Universal IFN than VP24, as NS5 allowed for virus titre to increase by 10^2.1^ TCID_50_/mL (p< 0.01) (Fig 4B). Thus, the NS5 protein of KFDV appears to be a potent inhibitor of the antiviral effects afforded by IFN.

## Discussion

As a treatment in clinical settings against flavivirus diseases, IFN-α2a and IFN-α2b have had variable success. When compared against the IFN-sensitive VSV-GFP virus, we discovered that IFN-α2a was unable to reduce KFDV replication in cell culture. Although some IFN subtypes appeared to be more potent than IFN-α2a at reducing KFDV replication, further analysis indicated that regardless of IFN subtype, IFN was inadequate against KFDV replication. It also appears that KFDV-induced CPE prevention is an unreliable criterion for the inhibitory effects of IFN, as the extent of CPE production did not coincide with the impact of IFN on viral titres. Furthermore, the minimized impact of IFN on KFDV to be an inherent ability of the virus correlated with activity of the NS5 protein.

For several flavivirus cell culture models, the antiviral potency of IFN decreases significantly once flavivirus infection has been established, especially after 4–6 hpi [25,31,40,43,53]. The finding that treatment at 1 hpi was more potent at reducing both VSV-GFP and KFDV than treatment at 24 hours pre-infection may be an A549 cellular-dependent observation. It is well known that the induction of the cellular antiviral program and duration of response (IFN and ISG expression) can vary with cell type [23]. With this in mind, another unrelated cell type BHK-21, which like A549 can respond to IFN-signalling, but is a weak IFN producer [48,54], was added into the virus clearance assay. In both cell types it was clear that IFN failed to quell KFDV infection. However since the infected cells were passaged, it may be possible that the passaged cells used for seeding new wells have been irreversibly compromised from the anti-IFN actions of KFDV. Thus, despite the cells’ ability to replicate and become confluent, they could have remained in a state that permitted KFDV infection, rendering IFN ineffective. Although the latter experiments indicate that NS5 can interfere with IFN signalling, its duration has not been investigated. Another interesting observation is the lack of CPE despite high titres of KFDV. While it is not clear why this is occurring, other flaviviruses promote autophagy induction to prolong cell survival and prevent apoptosis for long-term replication within autophagy compartments [55–57]. Additionally, the inhibition of apoptosis using a steroid (dehydroepiandrosterone) could prevent CPE during infection with Japanese encephalitis virus without impacting virus replication [58]. Perhaps KFDV is inhibiting apoptosis or utilizing autophagy to prevent apoptosis in the presence IFN and allowing apoptosis in the absence of IFN, as depicted in the differences in CPE development. Further studies should be conducted to confirm either of these hypotheses.

Initial screening of IFN-α/β subtypes suggested that IFN-αWA and IFN-αK were more potent than IFN-α2a at preventing KFDV virus production. While it is still unknown why these α and β subtypes display such a range in antiviral activity, it may be due to the strength of receptor binding or IFN-subtypes specific antiviral ISG expression [59,60]. It has been reported that virus concentration can impact distinct induction of not only ISG expression but also IFN-α subtype induction [61]. Perhaps, the potency of IFN-αWA and IFN-αK against the high MOI of KFDV challenge virus caused the expression of unique ISG profiles and should be further investigated for tick-borne flavivirus-specific ISGs. TRIM79α is a perfect example of a potent ISG that prevents infections by tick-borne but not mosquito-borne flaviviruses [62].

More in depth analysis of the IFN subtypes revealed that regardless of the concentration or subtypes applied, IFN may provide some protection from CPE but has very limited activity against KFDV replication in cell culture. This was evident when the more potent IFN subtypes (IFN-αWA and IFN-αK) were comparable at restricting KFDV replication to IFN-α2a at the lower MOI. This contrasting result is likely due to the fact that the MOI used influences the cellular ISG expression profiles [61]. In this case, the lower MOI may have reflected this difference in the ability of IFN-αWA and IFN-αK to induce anti-KFDV ISGs as observed from the higher MOI utilized in the screening assay.

Despite its traditional use for measuring IFN potency, the dye-uptake assay demonstrated that CPE prevention is not a valid measure for IFN potency against KFDV. Thus, any sort of CPE or cell viability assay for IFN potency against KFDV and perhaps other flaviviruses must be carefully considered, as the most accurate measurement proved to be determination of the number/concentration of infectious virus particles. An example of such a discrepancy was seen in Alkhumra hemorrhagic fever studies treated with IFN-α2a, as the concentrations required to reduced infection by 50% differed from 684 (+/- 499) U/mL for CPE (metabolic-cell viability assay) and 12 (+/- 6) U/mL for virus titres (qRT-PCR) [63]. Although genetic-quantification is not the same as infectious particle counts, this still demonstrates the inconsistency seen with CPE-based assays. Moreover, the same metabolic assay revealed that KFDV infection could be restricted by 50% infection with 863 (+/- 450) U/mL IFN-α2a. Unfortunately this was not confirmed by virus titre [63]. In another study, IC_50_ was defined by the visual-determination CPE reduction and IC_90_ was determined by reductions in virus titres. Viruses such as: dengue virus 1 had IC_50_ and IC_90_ values of 15.8 and 1 U/mL respectively and Zika virus had similar IC_50_ and IC_90_ values of 34 and 30 U/mL respectively [42]. In summation, it would appear that the most accurate measurement of IFN potency would infectious particle release. Our data indicate that IFN does not significantly reduce KFDV replication and virion production.

It appears that the KFDV NS5 protein plays an important role in the failure of IFN-α/β subtypes to control KFDV propagation. Further experimentation with knockdown assays was not feasible because the NS5 protein is critical for virus replication and our previous mutational analysis indicated that the anti-Jak/STAT pathway activity of NS5 was within the RNA-dependent RNA polymerase domain [28]. Aside from the Jak/STAT pathway it remains unclear which other IFN-induced pathways are targeted by the NS5 protein, allowing for recovery of the IFN-sensitive VSV-GFP virus in the presence of IFN. It would be interesting to examine further if KFDV much like other flaviviruses, can influence ISG production from not only the Jak/STAT pathway [28], but the phosphatidylinositol 3-kinase (PI3K), cyclic GMP-AMP synthase (cGAS) [64] and IRF-1 [65] pathways.

Flaviviruses respond to IFN very differently within the mosquito-borne and tick-borne groups and, even within strains or serotypes. IFN concentrations as low as 1 U/mL and as high as 200 U/mL have been successful at reducing *in vitro* infection from many flaviviruses by 90% [42]. Unlike these flaviviruses, KFDV infection was not limited by 90% using any of the IFN-α/β subtypes. Moreover, case reports indicate that IFN treatment can be marginal against flavivirus diseases and this is confounded by individuals who are immuno-compromised or those who delay or cannot readily access medical care [34–37]. While IFN does stimulate the adaptive immune response as summarised in [66], it is difficult to say how well KFDV infection would be controlled *in vivo* and should be investigated for confirmation. Nonetheless, the *in vitro* data presented here suggests that IFN may be an inadequate prophylaxis for KFDV infections and perhaps other treatment options should be explored.
